# (4,9-Dimethyl-9*H*-carbazol-3-yl)meth­anol

**DOI:** 10.1107/S1600536814003845

**Published:** 2014-02-22

**Authors:** Serkan Öncüoğlu, Nefise Dilek, Yavuz Ergün, Tuncer Hökelek

**Affiliations:** a Dokuz Eylül University, Faculty of Arts and Sciences, Department of Chemistry, Tınaztepe, 35160 Buca, İzmir, Turkey; b Aksaray University, Department of Physics, 68100, Aksaray, Turkey; c Hacettepe University, Department of Physics, 06800 Beytepe, Ankara, Turkey

## Abstract

In the title compound, C_15_H_15_NO, the carbazole skeleton includes a methanol group at the 3-position. The indole ring system is almost planar [maximum deviation = 0.045 (2) Å]. In the crystal, O—H⋯O hydrogen bonds link the mol­ecules into zigzag chains along the *b*-axis direction. There are weak C—H⋯π inter­actions within the chains and linking neighbouring chains forming sheets lying parallel to (001).

## Related literature   

For biological activity of carbazole alkaloids, see: Chakraborty (1977 ▶). For anti­biotic, anti­fungal and cytotoxic properties of carbazole alkaloids, see: Chakraborty *et al.* (1965 ▶); Chakraborty *et al.* (1978 ▶). For the use of carbazole derivatives as precursor compounds for the syntheses of pyridocarbazole alkaloids, see: Karmakar *et al.* (1991 ▶). For related structures, see: Hökelek *et al.* (1994 ▶); Patır *et al.* (1997 ▶); Öncüoğlu *et al.* (2014 ▶). For bond-length data, see: Allen *et al.* (1987 ▶).
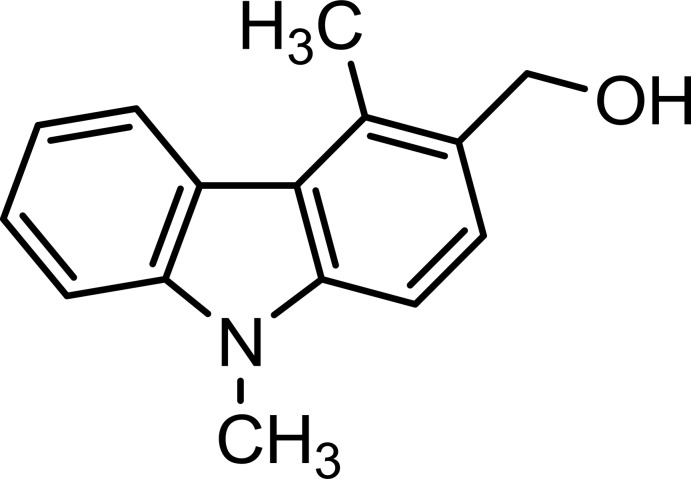


## Experimental   

### 

#### Crystal data  

C_15_H_15_NO
*M* *_r_* = 225.28
Monoclinic, 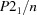

*a* = 14.4728 (4) Å
*b* = 5.4554 (3) Å
*c* = 15.0906 (4) Å
β = 95.453 (4)°
*V* = 1186.08 (8) Å^3^
*Z* = 4
Mo *K*α radiation
μ = 0.08 mm^−1^
*T* = 296 K
0.45 × 0.36 × 0.13 mm

#### Data collection  

Bruker SMART BREEZE CCD diffractometer
Absorption correction: multi-scan (*SADABS*; Bruker, 2007 ▶) *T*_min_ = 0.965, *T*_max_ = 0.990
11615 measured reflections
11615 independent reflections
9784 reflections with *I* > 2σ(*I*)
*R*_int_ = 0.032

#### Refinement  

*R*[*F* ^2^ > 2σ(*F* ^2^)] = 0.079
*wR*(*F* ^2^) = 0.214
*S* = 1.16
11615 reflections
161 parameters
H atoms treated by a mixture of independent and constrained refinement
Δρ_max_ = 0.30 e Å^−3^
Δρ_min_ = −0.27 e Å^−3^

### 

Data collection: *APEX2* (Bruker, 2007 ▶); cell refinement: *SAINT* (Bruker, 2007 ▶); data reduction: *SAINT*; program(s) used to solve structure: *SHELXS97* (Sheldrick, 2008 ▶); program(s) used to refine structure: *SHELXL97* (Sheldrick, 2008 ▶); molecular graphics: *ORTEP-3 for Windows* (Farrugia, 2012 ▶); software used to prepare material for publication: *WinGX* (Farrugia, 2012 ▶) and *PLATON* (Spek, 2009 ▶).

## Supplementary Material

Crystal structure: contains datablock(s) I, global. DOI: 10.1107/S1600536814003845/su2701sup1.cif

Structure factors: contains datablock(s) I. DOI: 10.1107/S1600536814003845/su2701Isup2.hkl

Supporting information file. DOI: 10.1107/S1600536814003845/su2701Isup3.cml

CCDC reference:

Additional supporting information:  crystallographic information; 3D view; checkCIF report

## Figures and Tables

**Table 1:** Hydrogen-bond geometry (Å, °) *Cg*1 and *Cg*2 are the centroids of rings 9a/C1-C4/C4a/ and C5a/C5-C8/C8a, respectively.

*D*—H⋯*A*	*D*—H	H⋯*A*	*D*⋯*A*	*D*—H⋯*A*
O1—H1*A*⋯O1^i^	0.88 (3)	2.13 (3)	2.919 (2)	149 (3)
C10—H10*A*⋯*Cg*2^ii^	0.96	2.85	3.697 (2)	148
C10—H10*B*⋯*Cg*1^iii^	0.96	2.64	3.531 (2)	154
C11—H11*A*⋯*Cg*2^iv^	0.96	2.77	3.617 (2)	147

Symmetry codes: (i) 
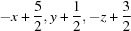
; (ii) 
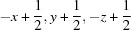
; (iii) 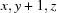; (iv) 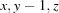.

**Fig. 1.**
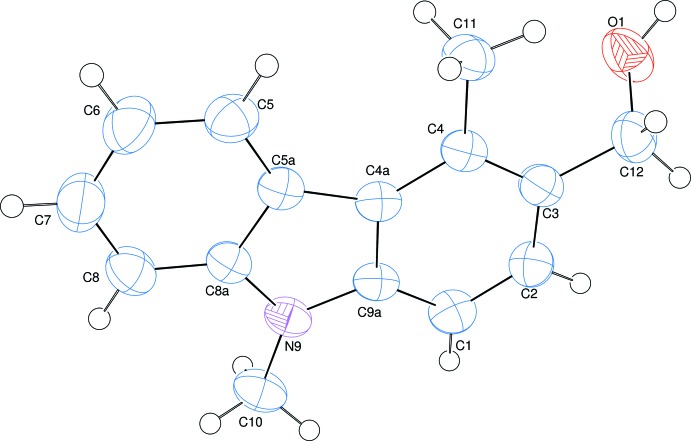
The molecular structure of the title molecule, with atom labelling. Displacement ellipsoids are drawn at the 50% probability level.

**Fig. 2.**
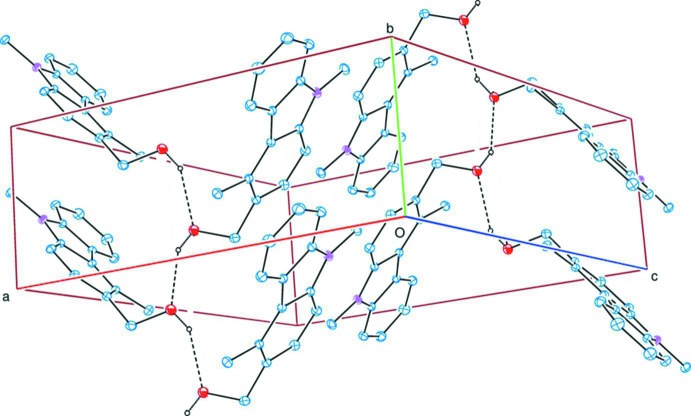
A view of the crystal packing of the title compound with the O-H···O hydrogen bonds shown as dashed lines (see Table 1 for details; H atoms not involved in hydrogen bonding have been omitted for clarity].

## supplementary crystallographic information

### 1. Comment

Carbazole alkaloids, which have their richest source in species of the genus *Murraya*, are of great interest because of their unique structures and important biological activities (Chakraborty, 1977). They also exhibits antibiotic, antifungal and cytotoxic properties (Chakraborty *et al.*, 1965; Chakraborty *et al.*, 1978). Carbazole derivatives are also used as precursor compounds for the syntheses of pyridocarbazole alkaloids (Karmakar *et al.*, 1991). The present study was undertaken to ascertain the crystal structure of the title compound which was first synthesized by (Karmakar *et al.*, 1991).

The molecule of the title compound contains a carbazole skeleton with a methanol group at the 3 position, Fig. 1. The bond lengths are close to standard values (Allen *et al.*, 1987) and generally agree with those in previously reported compounds (Hökelek *et al.*, 1994; Patır *et al.*, 1997; Öncüoğlu *et al.*, 2014). In all structures atom N9 is substituted.

An examination of the deviations from the mean planes through individual rings shows that rings *A* (C1—C4/C4a/c9a), *B* (C4a/C5a/C8a/N9/C9a) and *C* (C5a/C5—C8/C8a) are nearly coplanar [with a maximum deviation of 0.045 (2) Å for atom C7] with dihedral angles of A/B = 0.76 (5), A/C = 2.33 (4) and B/C = 1.57 (5) °. Atoms C10, C11 and C12 are displaced by 0.070 (2), 0.004 (2) and -0.025 (2) Å from the adjacent ring planes.

In the crystal, O—H···O hydrogen bonds link the molecules into zigzag chains along the *b*-axis direction (Table 1 and Fig. 2). There are weak C—H···π interactions within the chains and linking neighbouring chains forming two-dimensional networks lying parallel to (001); see Table 1.

### 2. Experimental

The title compound was synthesized according to the literature method (Karmakar *et al.*, 1991). A solution of ethyl 4,9-dimethyl-9*H*-carbazole-3 -carboxylate (4.00 g, 15 mmol) in anhydrous tetrahydrofurane (50 ml) was added drop wise to a stirred solution of lithium aluminium hydride (1.20 g, 31 mmol) in tetrahydrofurane at room temperature. The reaction mixture was refluxed for 5 h under a nitrogen atmosphere, and then cooled and the excess of lithium aluminium hydride was destroyed with water and extracted with ethyl acetate. The organic phase was dried with anhydrous magnesium sulfate, and the solvent was evaporated. The crude product was recrystallized from ether (Yield; 95%, M.p. 475 K), giving block-like colourless crystals suitable for X-ray diffraction analysis.

### 3. Refinement

Atom H1A (for OH) was located in a difference Fourier map and freely refined. The C-bound H-atoms were positioned geometrically with C—H = 0.93, 0.97 and 0.96 Å, for aromatic, methylene and methyl H-atoms, respectively, and constrained to ride on their parent atoms with *U*_iso_(H) = 1.5*U*_eq_(C-methyl) and = 1.2U_eq_(C) for other H-atoms.

### Crystal data

**Table:**

C_15_H_15_NO	*F*(000) = 480
*M**_r_* = 225.28	*D*_x_ = 1.262 Mg m^−^^3^
Monoclinic, *P*2_1_/*n*	Mo *K*α radiation, λ = 0.71073 Å
Hall symbol: -P 2yn	Cell parameters from 6741 reflections
*a* = 14.4728 (4) Å	θ = 2.7–28.2°
*b* = 5.4554 (3) Å	µ = 0.08 mm^−^^1^
*c* = 15.0906 (4) Å	*T* = 296 K
β = 95.453 (4)°	Block, colourless
*V* = 1186.08 (8) Å^3^	0.45 × 0.36 × 0.13 mm
*Z* = 4	

### Data collection

**Table:**

Bruker SMART BREEZE CCD diffractometer	11615 independent reflections
Radiation source: fine-focus sealed tube	9784 reflections with *I* > 2σ(*I*)
Graphite monochromator	*R*_int_ = 0.032
φ and ω scans	θ_max_ = 26.4°, θ_min_ = 1.9°
Absorption correction: multi-scan (*SADABS*; Bruker, 2007)	*h* = −18→17
*T*_min_ = 0.965, *T*_max_ = 0.990	*k* = −6→6
11615 measured reflections	*l* = −18→18

### Refinement

**Table:**

Refinement on *F*^2^	Primary atom site location: structure-invariant direct methods
Least-squares matrix: full	Secondary atom site location: difference Fourier map
*R*[*F*^2^ > 2σ(*F*^2^)] = 0.079	Hydrogen site location: inferred from neighbouring sites
*wR*(*F*^2^) = 0.214	H atoms treated by a mixture of independent and constrained refinement
*S* = 1.16	*w* = 1/[σ^2^(*F*_o_^2^) + (0.0293*P*)^2^ + 3.1387*P*] where *P* = (*F*_o_^2^ + 2*F*_c_^2^)/3
11615 reflections	(Δ/σ)_max_ < 0.001
161 parameters	Δρ_max_ = 0.30 e Å^−^^3^
0 restraints	Δρ_min_ = −0.27 e Å^−^^3^

### Special details

**Table:**

Geometry. All esds (except the esd in the dihedral angle between two l.s. planes) are estimated using the full covariance matrix. The cell esds are taken into account individually in the estimation of esds in distances, angles and torsion angles; correlations between esds in cell parameters are only used when they are defined by crystal symmetry. An approximate (isotropic) treatment of cell esds is used for estimating esds involving l.s. planes.
Refinement. Refinement of F^2^ against ALL reflections. The weighted R-factor wR and goodness of fit S are based on F^2^, conventional R-factors R are based on F, with F set to zero for negative F^2^. The threshold expression of F^2^ > 2sigma(F^2^) is used only for calculating R-factors(gt) etc. and is not relevant to the choice of reflections for refinement. R-factors based on F^2^ are statistically about twice as large as those based on F, and R- factors based on ALL data will be even larger.

### Fractional atomic coordinates and isotropic or equivalent isotropic displacement parameters (Å^2^)

**Table:**

	*x*	*y*	*z*	*U*_iso_*/*U*_eq_	
O1	1.23676 (10)	1.1637 (3)	0.78082 (11)	0.0647 (4)	
H1A	1.262 (2)	1.285 (6)	0.7532 (18)	0.115 (11)*	
C1	1.00323 (13)	0.7254 (3)	0.89293 (11)	0.0435 (4)	
H1	0.9915	0.6531	0.9465	0.052*	
C2	1.06557 (13)	0.9145 (3)	0.89012 (11)	0.0449 (4)	
H2	1.0966	0.9690	0.9433	0.054*	
C3	1.08440 (12)	1.0289 (3)	0.81060 (11)	0.0416 (4)	
C4	1.03968 (12)	0.9504 (3)	0.72954 (11)	0.0379 (4)	
C4A	0.97556 (12)	0.7560 (3)	0.73093 (10)	0.0361 (4)	
C5	0.90173 (14)	0.6439 (4)	0.56991 (11)	0.0503 (5)	
H5	0.9309	0.7656	0.5396	0.060*	
C5A	0.91717 (12)	0.6257 (3)	0.66236 (10)	0.0377 (4)	
C6	0.84282 (16)	0.4795 (4)	0.52398 (13)	0.0604 (6)	
H6	0.8332	0.4883	0.4622	0.072*	
C7	0.79780 (15)	0.3012 (4)	0.56927 (13)	0.0612 (6)	
H7	0.7582	0.1924	0.5370	0.073*	
C8	0.80997 (14)	0.2801 (3)	0.66092 (13)	0.0510 (5)	
H8	0.7793	0.1603	0.6908	0.061*	
C8A	0.86991 (12)	0.4451 (3)	0.70644 (11)	0.0381 (4)	
N9	0.89428 (10)	0.4597 (3)	0.79697 (9)	0.0404 (4)	
C9A	0.95842 (12)	0.6465 (3)	0.81262 (10)	0.0368 (4)	
C10	0.86281 (14)	0.2951 (3)	0.86346 (12)	0.0506 (5)	
H10B	0.9017	0.1524	0.8682	0.076*	
H10A	0.7999	0.2470	0.8462	0.076*	
H10C	0.8660	0.3772	0.9199	0.076*	
C11	1.05756 (14)	1.0659 (3)	0.64152 (11)	0.0507 (5)	
H11B	1.0828	0.9451	0.6043	0.076*	
H11C	1.1009	1.1984	0.6519	0.076*	
H11A	1.0003	1.1273	0.6125	0.076*	
C12	1.15214 (14)	1.2370 (3)	0.81458 (13)	0.0532 (5)	
H12A	1.1254	1.3734	0.7798	0.064*	
H12B	1.1648	1.2913	0.8757	0.064*	

### Atomic displacement parameters (Å^2^)

**Table:**

	*U*^11^	*U*^22^	*U*^33^	*U*^12^	*U*^13^	*U*^23^
O1	0.0439 (9)	0.0484 (9)	0.1046 (12)	−0.0001 (7)	0.0218 (8)	−0.0003 (8)
C1	0.0526 (12)	0.0403 (10)	0.0373 (9)	0.0063 (9)	0.0031 (8)	0.0124 (7)
C2	0.0461 (11)	0.0471 (11)	0.0405 (10)	0.0013 (9)	−0.0008 (8)	0.0021 (7)
C3	0.0398 (11)	0.0368 (10)	0.0490 (10)	0.0027 (8)	0.0091 (8)	0.0026 (7)
C4	0.0374 (10)	0.0329 (9)	0.0441 (9)	0.0073 (7)	0.0081 (7)	0.0061 (7)
C4A	0.0376 (10)	0.0352 (9)	0.0360 (8)	0.0067 (7)	0.0059 (7)	0.0068 (6)
C5	0.0604 (13)	0.0480 (11)	0.0433 (10)	0.0013 (10)	0.0085 (9)	0.0067 (8)
C5A	0.0364 (10)	0.0342 (9)	0.0431 (9)	0.0068 (7)	0.0063 (7)	0.0037 (7)
C6	0.0760 (16)	0.0627 (14)	0.0408 (10)	−0.0055 (12)	−0.0029 (10)	−0.0011 (9)
C7	0.0618 (14)	0.0605 (14)	0.0599 (13)	−0.0065 (11)	−0.0013 (10)	−0.0118 (10)
C8	0.0517 (13)	0.0423 (11)	0.0597 (12)	−0.0045 (9)	0.0100 (9)	0.0060 (8)
C8A	0.0349 (10)	0.0352 (9)	0.0445 (9)	0.0034 (7)	0.0064 (7)	0.0042 (7)
N9	0.0416 (9)	0.0389 (8)	0.0417 (8)	0.0001 (7)	0.0086 (6)	0.0118 (6)
C9A	0.0387 (10)	0.0335 (9)	0.0394 (9)	0.0071 (8)	0.0097 (7)	0.0085 (7)
C10	0.0524 (12)	0.0474 (11)	0.0537 (11)	0.0000 (9)	0.0136 (9)	0.0207 (8)
C11	0.0563 (13)	0.0472 (11)	0.0502 (11)	−0.0036 (9)	0.0135 (9)	0.0081 (8)
C12	0.0525 (13)	0.0382 (11)	0.0697 (13)	−0.0006 (9)	0.0107 (10)	−0.0037 (9)

### Geometric parameters (Å, º)

**Table:**

O1—C12	1.427 (2)	C7—H7	0.9300
O1—H1A	0.88 (3)	C8—C7	1.382 (3)
C1—C2	1.374 (3)	C8—H8	0.9300
C1—H1	0.9300	C8A—C8	1.386 (3)
C2—C3	1.402 (2)	N9—C8A	1.380 (2)
C2—H2	0.9300	N9—C9A	1.383 (2)
C3—C12	1.497 (3)	N9—C10	1.4517 (19)
C4—C3	1.396 (2)	C9A—C1	1.387 (2)
C4—C4A	1.411 (2)	C9A—C4A	1.413 (2)
C4—C11	1.514 (2)	C10—H10A	0.9600
C5—C6	1.378 (3)	C10—H10B	0.9600
C5—H5	0.9300	C10—H10C	0.9600
C5A—C4A	1.457 (2)	C11—H11A	0.9600
C5A—C5	1.395 (2)	C11—H11B	0.9600
C5A—C8A	1.403 (2)	C11—H11C	0.9600
C6—C7	1.387 (3)	C12—H12A	0.9700
C6—H6	0.9300	C12—H12B	0.9700
			
C12—O1—H1A	111.3 (19)	C7—C8—H8	121.4
C2—C1—C9A	117.38 (15)	C8A—C8—H8	121.4
C2—C1—H1	121.3	N9—C8A—C8	128.11 (15)
C9A—C1—H1	121.3	N9—C8A—C5A	109.77 (15)
C1—C2—C3	122.84 (16)	C8—C8A—C5A	122.11 (16)
C1—C2—H2	118.6	C8A—N9—C9A	108.42 (12)
C3—C2—H2	118.6	C8A—N9—C10	125.50 (15)
C2—C3—C12	118.88 (17)	C9A—N9—C10	125.95 (14)
C4—C3—C2	120.12 (16)	N9—C9A—C1	128.90 (14)
C4—C3—C12	121.00 (15)	N9—C9A—C4A	109.46 (14)
C3—C4—C4A	117.94 (14)	C1—C9A—C4A	121.64 (16)
C3—C4—C11	122.51 (16)	N9—C10—H10A	109.5
C4A—C4—C11	119.55 (15)	N9—C10—H10B	109.5
C4—C4A—C5A	133.96 (14)	N9—C10—H10C	109.5
C4—C4A—C9A	120.08 (15)	H10A—C10—H10C	109.5
C9A—C4A—C5A	105.96 (15)	H10B—C10—H10A	109.5
C5A—C5—H5	120.4	H10B—C10—H10C	109.5
C6—C5—C5A	119.29 (18)	C4—C11—H11A	109.5
C6—C5—H5	120.4	C4—C11—H11B	109.5
C5—C5A—C4A	134.60 (16)	C4—C11—H11C	109.5
C5—C5A—C8A	119.02 (16)	H11B—C11—H11A	109.5
C8A—C5A—C4A	106.38 (14)	H11B—C11—H11C	109.5
C5—C6—C7	120.39 (18)	H11C—C11—H11A	109.5
C5—C6—H6	119.8	O1—C12—C3	110.74 (15)
C7—C6—H6	119.8	O1—C12—H12A	109.5
C6—C7—H7	119.0	O1—C12—H12B	109.5
C8—C7—C6	122.03 (19)	C3—C12—H12A	109.5
C8—C7—H7	119.0	C3—C12—H12B	109.5
C7—C8—C8A	117.13 (17)	H12A—C12—H12B	108.1
			
C9A—C1—C2—C3	0.5 (3)	C4A—C5A—C8A—C8	−177.86 (16)
C1—C2—C3—C4	−0.6 (3)	C5—C5A—C8A—N9	−179.48 (15)
C1—C2—C3—C12	178.80 (17)	C5—C5A—C8A—C8	1.6 (3)
C2—C3—C12—O1	107.98 (19)	C5—C6—C7—C8	−0.2 (3)
C4—C3—C12—O1	−72.7 (2)	C8A—C8—C7—C6	−0.3 (3)
C4A—C4—C3—C2	0.4 (2)	N9—C8A—C8—C7	−179.16 (17)
C4A—C4—C3—C12	−178.98 (16)	C5A—C8A—C8—C7	−0.4 (3)
C11—C4—C3—C2	−179.65 (16)	C9A—N9—C8A—C5A	−0.94 (18)
C11—C4—C3—C12	1.0 (3)	C9A—N9—C8A—C8	177.93 (18)
C3—C4—C4A—C5A	−179.64 (17)	C10—N9—C8A—C5A	−176.86 (16)
C3—C4—C4A—C9A	−0.1 (2)	C10—N9—C8A—C8	2.0 (3)
C11—C4—C4A—C5A	0.4 (3)	C8A—N9—C9A—C1	−178.98 (17)
C11—C4—C4A—C9A	179.87 (15)	C8A—N9—C9A—C4A	0.39 (18)
C5A—C5—C6—C7	1.4 (3)	C10—N9—C9A—C1	−3.1 (3)
C5—C5A—C4A—C4	−0.6 (3)	C10—N9—C9A—C4A	176.28 (15)
C5—C5A—C4A—C9A	179.88 (19)	N9—C9A—C1—C2	179.05 (17)
C8A—C5A—C4A—C4	178.71 (17)	C4A—C9A—C1—C2	−0.2 (3)
C8A—C5A—C4A—C9A	−0.83 (18)	N9—C9A—C4A—C4	−179.33 (14)
C4A—C5A—C5—C6	177.21 (19)	N9—C9A—C4A—C5A	0.29 (18)
C8A—C5A—C5—C6	−2.0 (3)	C1—C9A—C4A—C4	0.1 (2)
C4A—C5A—C8A—N9	1.10 (18)	C1—C9A—C4A—C5A	179.70 (15)

### Hydrogen-bond geometry (Å, º)

Cg1 and Cg2 are the centroids of rings 9a/C1-C4/C4a/ and C5a/C5-C8/C8a, respectively.

**Table:**

*D*—H···*A*	*D*—H	H···*A*	*D*···*A*	*D*—H···*A*
O1—H1*A*···O1^i^	0.88 (3)	2.13 (3)	2.919 (2)	149 (3)
C10—H10*A*···*Cg*2^ii^	0.96	2.85	3.697 (2)	148
C10—H10*B*···*Cg*1^iii^	0.96	2.64	3.531 (2)	154
C11—H11*A*···*Cg*2^iv^	0.96	2.77	3.617 (2)	147

Symmetry codes: (i) −*x*+5/2, *y*+1/2, −*z*+3/2; (ii) −*x*+1/2, *y*+1/2, −*z*+1/2; (iii) *x*, *y*+1, *z*; (iv) *x*, *y*−1, *z*.
